# The Vaccination Papers of Practical and Immediate Value

**Published:** 1898-06

**Authors:** F. Dobson

**Affiliations:** Brooklyn, N. Y.


### THE VACCINATION PAPERS OF PRACTICAL AND IMMEDIATE VALUE.

BROOKLYN, N. Y., May 7th, 1898.

EDITOR OF THE HOMŒOPATHIC PHYSICIAN:—In response to your invitation in March number of HOMŒOPATHIC PHYSICIAN, kindly permit me to say that Dr. Leverson's abstracts are of practical and of immediate value in a matter of the highest importance to the homœopathic profession and the public.

They are proving a valuable educational force, and will in the future be of even more force than now.

The vaccinal theory in regard to the so-called germ and zymotic diseases is receiving wide attention at the hands of the old school, and, I regret to say, that many of the homœopathic profession are deluded by the specious pleas put forth. Especially is this the case in "Pasteurism," and more particularly with "Anti-toxins" for hydrophobia and diphtheria.

F. Dobson, M. D.

